# The Effects of High-fat-diet Combined with Chronic Unpredictable Mild Stress on Depression-like Behavior and Leptin/LepRb in Male Rats

**DOI:** 10.1038/srep35239

**Published:** 2016-10-14

**Authors:** Jin Ling Yang, De Xiang Liu, Hong Jiang, Fang Pan, Cyrus SH Ho, Roger CM Ho

**Affiliations:** 1 Department of Medical Psychology, Shandong University School of Medicine, Jinan, Shandong, 250012, China; 2 Department of Psychological Medicine, National University of Singapore, Singapore, 119228

## Abstract

Leptin plays a key role in the pathogenesis of obesity and depression via the long form of leptin receptor (LepRb). An animal model of comorbid obesity and depression induced by high-fat diet (HFD) combined with chronic unpredictable mild stress (CUMS) was developed to study the relationship between depression/anxiety-like behavior, levels of plasma leptin and LepRb in the brains between four groups of rats, the combined obesity and CUMS (Co) group, the obese (Ob) group, the CUMS group and controls. Our results revealed that the Co group exhibited most severe depression-like behavior in the open field test (OFT), anxiety-like behavior in elevated plus maze test (EMT) and cognitive impairment in the Morris water maze (MWM). The Ob group had the highest weight and plasma leptin levels while the Co group had the lowest levels of protein of LepRb in the hypothalamus and hippocampus. Furthermore, depressive and anxiety-like behaviors as well as cognitive impairment were positively correlated with levels of LepRb protein and mRNA in the hippocampus and hypothalamus. The down-regulation of leptin/LepRb signaling might be associated with depressive-like behavior and cognitive impairment in obese rats facing chronic mild stress.

Obesity is a condition characterized by massive adipose tissue accumulation, which is usually caused by complicated genetic and environmental factors^1^,^2^,^3^. Obesity is associated with many health problems including metabolic syndrome, cardiovascular diseases and psychiatric disorders^4^,^5^,^6^,^7^. Depression is a major psychiatric disorder that is characterized by low mood, anhedonia and cognitive impairment^8^,^9^. Clinical and epidemiological data suggest that obesity and depression frequently coexist^10^,^11^ and obesity is associated with an increased risk of depressive disorder^12^. Comorbid obesity and depression contribute to significant morbidity^13^,^14^ and functional impairment^15^.

The long-term excessive intake of calories is regarded as a risk factor for obesity. Using high fat diet (HFD) to induce obesity is an established animal model of obesity which causes excessive body weight, higher adipose/weight ratio and higher serum levels of free fatty acid^16^,^17^. Meanwhile, the chronic unexpected mild stress (CUMS) model has been widely used as a highly predictive and valid animal model of depression^18^. Long-term uncontrollable and unpredictable stressors will result in depression-like and anxiety-like symptoms in animals^19^ with impairments in spatial learning and memory. These behavioral and cognitive changes could be examined by behavioral tests. Depression-like behavior such as anhedonia and deficit of explorative ability are evaluated by the sucrose preference tests (SPT) and open field test (OPT) respectively. Anxiety-like behavior is assessed by elevated plus maze test (EPT) and spatial learning can be evaluated by using the Morris water maze (MWM) test.

Leptin is a hormone derived from adipose tissue that provides feedback about adiposity to the brain^20^. It is circulated in blood, crosses the blood-brain barrier and exerts its effects on the leptin receptor, which are expressed in the hypothalamus, hippocampus and cerebral cortex^21^. The long form of leptin receptor (LepRb) mediates the biological actions of leptin and leads to intracellular signal transduction. Obese individuals having elevated plasma leptin level is known as developing leptin resistance^22^. When the adiposity to weight ratio is high, leptin decreases food intake and increases energy expenditure through the action of LepRb in the hypothalamus^23^. Leptin and LepRb were also found to play a role in the pathogenesis of depressive disorder. Some studies found that depressed patients with normal adiposity had high leptin levels^24^,^25^. In contrast, other studies found that low leptin levels were associated with depression^26^,^27^ and injection of leptin into hippocampus had antidepressant effects^28^. Milaneschi *et al*. demonstrated that the association between leptin and depressive symptoms was modulated by abdominal adiposity in humans but it was not possible to study the role of LepRb in humans^29^. These inconsistent findings suggest that other factors such as obesity might influence the interaction between depression and leptin/LepRb. The relationship between leptin/LepRb, obesity and depression requires further study.

The hippocampus has been suggested to be involved in the pathophysiology of cognitive impairment in patients suffering from depressive disorder. The hypothalamus is affected by stress and depression through the neuroendocrine system^30^. In this study, one group of rats was fed by the HFD for 8 weeks to induce obesity followed by 3 weeks of CUMS and HFD to establish an animal model of comorbid obesity and depression. Behavioral tests were performed to assess the emotional and cognitive disturbances in rats. The leptin/LepRb signaling was tested subsequently to identify the levels of plasma leptin and expression of protein and mRNA of LepRb in the hippocampus and hypothalamus and their relationship with weight gain, depression/anxiety-like behavior and cognitive impairment in rats that were exposed to HFD and CUMS, CUMS only, HFD only and the control group.

## Results

### The Effects of HFD and CUMS on body weight and adipose/body weight

**Figure 1:**
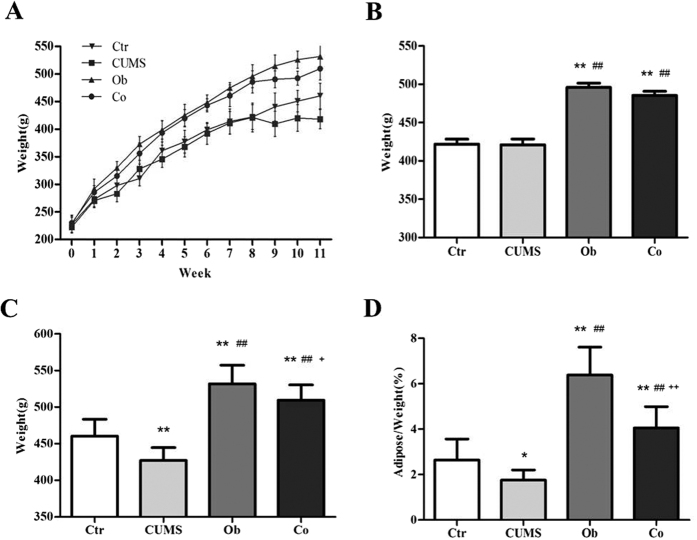
The body weight and adipose/weight of the 4 groups. (**A**) The change in body weight in the HFD and CUMS groups. (**B**) Average body weight at the 8^th^ week; (**C**) Average body weight at the 11^th^ week; (**D**) Adipose/Weight at the 11^th^ week. n = 12 in the Ctr and CUMS groups respectively, n = 13 in Ob and Co groups respectively. All data are expressed as means ± SD; *P < 0.05, **P < 0.01 vs. Ctr group; ^##^*P* < 0.01 vs. CUMS group; ^+^*P* < 0.05 vs. Ob group, ^++^*P *< 0.01 vs. Ob group.

The changes in body weight of different groups are shown in Fig. 1A. The results show that consumption of the HFD induced higher body weight gain in the HFD groups (i.e. the Ob and Co groups) compared with the regular diet (RD) groups (i.e. the CUMS and Ctr groups). The weight differences between the HFD groups and RD groups had increased gradually from the 1^st^ week to the 8^th^ week. The weight of the Ob group and the Co group remained higher than the CUMS and Ctr groups throughout the experiment. The CUMS procedure (from the 9^th^ to 11^th^ week) reduced the body weight in both CUMS and Co groups. In the 9^th^ week, which was the first week of CUMS procedure, the weight of the CUMS group showed decline from 421.1 ± 26.0 g to 409.5 ± 22.7 g while the Co group demonstrated mild increase in weight from 485.7 ± 20.1 g to 490.3 ± 15.3 g. Figure 1B shows significant differences in body weight among the 4 groups at the 8^th^ week [F (3, 46) = 41.364, p < 0.001]. Body weight of the Ob and Co groups were significantly higher than the Ctr group (p < 0.001, respectively) and CUMS group (p < 0.001, respectively). Figure 1C shows the difference in body weight among the 4 groups at the 11^th^ week [F (3, 46) = 40.291, p < 0.001]. There was no interaction between HFD and CUMS on body weight [F = 0.127, p = 0.723]. The CUMS group had lower body weight than the Ctr group (p < 0.001); The Co group had lower body weight than the Ob group (p < 0.05); The Ob and Co groups maintained higher body weight than the Ctr group (p < 0.001, respectively), Main effects of the HFD and CUMS were both significant [F = 105.462, p < 0.001; F = 15.372, p < 0.001] and indicated that 3 weeks of CUMS had caused significant reduction in the body weight while 11 weeks of HFD had caused significant increase in the body weight. Figure 1D shows that the adipose/weight among the 4 groups was significantly different [F (3, 46) = 59.295, p < 0.001]. The interaction between HFD and CUMS on adipose/weight was significant [F = 5.233, p < 0.05]. Main effects of the HFD and CUMS were significant [F = 139.450, p < 0.001; F = 31.808, p < 0.001]. The CUMS led to significantly lower adipose/weight in the Co group as compared with the Ob group (p < 0.001).

### The Effects of HFD and CUMS on depression-like behavior, anxiety-like behavior and cognitive impairment

**Figure 2:**
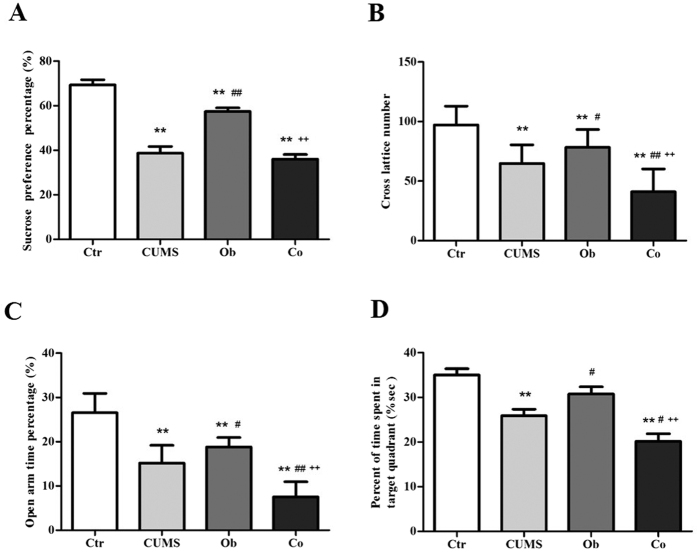
The effects of HFD and CUMS on the behavior tests at the 11^th^ week in the Ctr group (n = 12), CUMS group (n = 12), Ob group (n = 13) and Co group (n = 13). (**A**) Sucrose preference percentage; (**B**) Cross lattice number in the open-field test; (**C**) Time percentage spent in the open arm in the elevated plus-maze test; (**D**) percent of time in target quadrant in the Morris water maze. All data are expressed as means ± SD; *P < 0.05, **P < 0.01, vs. Ctr group; ^#^*P* < 0.05, ^##^*P* < 0.01, vs. CUMS group; ^++^p < 0.01, vs. Ob group.

Figure 2A illustrates results of the SPT [F (3, 46)  = 47.221, p < 0.001]. The interaction between HFD and CUMS had no effect on the SPT [F = 4.029, p = 0.051]. Main effects of the HFD and CUMS were significant [F = 10.050, p < 0.05; F = 129.199, p < 0.001]. The CUMS, Ob and Co groups had significantly lower sucrose preference than the Ctr group (p < 0.001, p < 0.05, p < 0.001 respectively). The CUMS and Co groups had lower level of sucrose preference than the Ob group (p < 0.001, respectively), indicating that the CUMS had worsen anhedonia in the CUMS and CO groups. Figure 2B shows the result of OFT [F (3, 46) = 25.569, p < 0.001]. Significant main effects of the HFD and CUMS on OFT were found [F = 20.669, p < 0.001; F = 55.363, p < 0.001], but the interaction between HFD and CUMS was not significant [F = 0.274, p = 0.603]. The CUMS, Ob and Co groups had lower cross lattice number than the Ctr group. The Co group had the lowest cross lattice number among the 4 groups, indicating that exposure to the CUMS and HFD significantly reduced automatic and exploring behavior. Figure 2C shows results of the EMT [F (3, 46) = 62.014, p = 0.000]. Main effects of the HFD and CUMS on EMT were significant [F = 58.573, p = 0.000; F = 127.308, p = 0.000], but the interaction between HFD and CUMS was not significant [F = 0.003, p = 0.958]. The CUMS, Ob and Co groups had lower percentage of time spent in the open arms than the Ctr group. The Co group had the lowest percentage of time spent in the open arms among the 4 groups, indicating exposure to the CUMS and HFD caused highest level of anxiety. Figure 2D displays the results of MWM [F (3, 46) = 16.904, p < 0.001]. Main effects of the HFD and CUMS were significant [F = 10.289, p < 0.05; F = 39.895, p < 0.001;], while interaction between HFD and CUMS was not significant [F = 0.224, p = 0.638]. The CUMS group spent lower percent of time in target quadrant than the Ctr group (p < 0.001). The Co group spent the lowest percent of time in target quadrant among the 4 groups (p < 0.001, p < 0.05, p < 0.001) which suggest exposure to the CUMS and HFD caused significant impairment in spatial learning and memory.

### The Effects of HFD and CUMS on plasma leptin level

**Figure 3:**
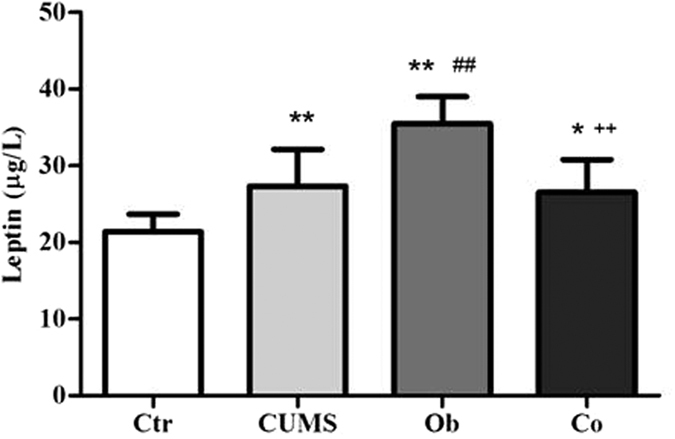
The plasma leptin (n = 8 per group). All data are expressed as means ± SD; *P < 0.05, **P < 0.01 vs. Ctr group; ^##^*P* < 0.01 vs. CUMS group; ^++^*P* < 0.01 vs. Ob group.

Figure 3 shows the levels of plasma leptin [F (3, 26) = 18.320, p = 0.000]. Main effect of the HFD on the levels of plasma leptin was significant [F = 23.680, p = 0.000], and the CUMS was not a significant factor affecting the levels of plasma leptin [F = 1.234, p = 0.276]. The interaction between HFD and CUMS on the levels of plasma leptin was significant [F = 30.046, p = 0.000]. The CUMS and Co groups had significantly higher levels of plasma leptin compared with the Ctr group (p = 0.004, p = 0.013). The Ob group had the highest levels of plasma leptin among the 4 groups (p = 0.000, respectively), indicating CUMS could reverse increased level of plasma leptin.

### The Effects of HFD and CUMS on LepRb protein

**Figure 4:**
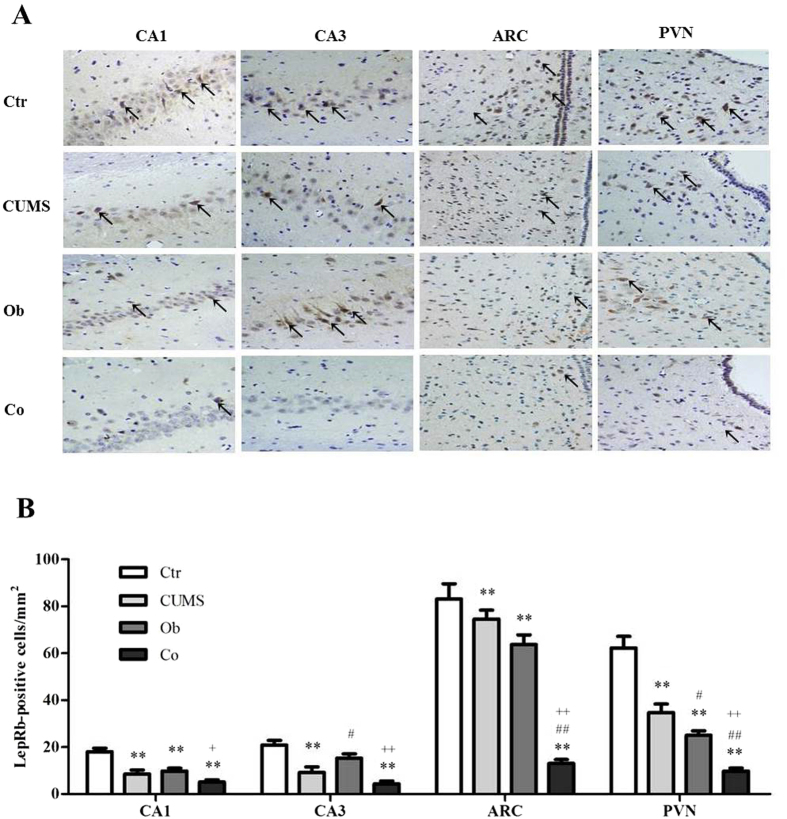
Graphical presentation of the expression of the LepRb protein in the hippocampus and hypothalamus by immunohistochemistry staining. (n = 4∼5 per group). (**A**) Immunohistochemistry staining of LepRb protein in the hippocampus (CA1 and CA3 subfields) and the hypothalamus (ARC and PVN subfields) (400×); (**B**) Data are expressed as means ± SD in each group; *P < 0.05, **P < 0.01 vs. Ctr group; ^#^*P* < 0.05, ^##^*P* < 0.01 vs. CUMS group; ^+^*P* < 0.05 vs. Ob group, ^++^*P* < 0.01 vs. Ob group.

Figure 4 shows the immunohistochemistry stain of the expression of LepRb protein (A) and data analysis of each group (B). The inmunohistochemistry stain of LepRb protein in the hippocampus and hypothalamus demonstrated brownish yellow granules in the cytoplasm and membrane of the LepRb positive neurons. Few expression of LepRb -positive neurons was observed in the CA1 region of the hippocampus in the CUMS, Ob and Co groups compared with the Ctr group (p = 0.000, respectively), as well as CA3 region of the hippocampus in the CUMS (p = 0.000) and Co (p = 0.000) groups as compared to the Ctr group, indicating that both HFD and CUMS exposure reduced the expression of LepRb in the hippocampus. In the hypothalamus, less expression of LepRb positive neurons was observed in the arcuate nucleus (ARC) and the paraventricular nucleus (PVN) regions in the CUMS (p = 0.047, p = 0.000), Ob (p = 0.005, p = 0.000) and Co (p = 0.000, p = 0.000) groups as compared with the Ctr group, suggesting that HFD and CUMS exposure reduced the expression of LepRb in the hypothalamus. These findings were confirmed in later analysis using the Western bolt and Real Time PCR.

**Figure 5:**
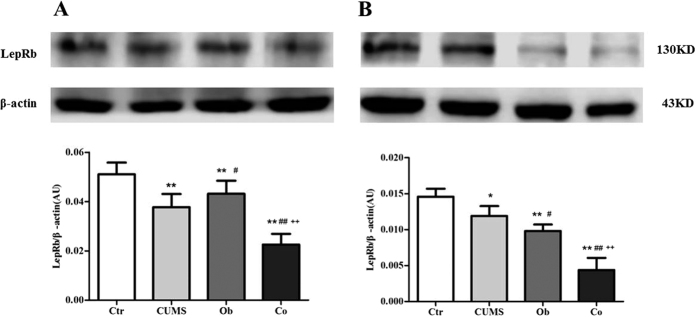
The expression of LepRb protein in the hippocampus (**A**) and hypothalamus (**B**) (n = 6 per group). All data are expressed as means ± SD; *P < 0.05, **P < 0.01 vs. Ctr group; ^#^*P* < .05 vs. CUMS group, ^##^*P* < 0.01 vs. CUMS group; ^+^*P* < 0.05 vs. Ob group, ^++^*P* < 0.01 vs. Ob group.

Figure 5A shows the expression of LepRb protein in the hippocampus by Western blot [F (3, 20) = 50.427, p = 0.000]. Main effects of the HFD on the expression of LepRb protein in the hippocampus were not significant [F = 0.021, p = 0.886], but main effect of stress on the expression of LepRb in the hippocampus was significant [F = 104.681, p = 0.000], and the interaction effect between HFD and CUMS was also significant [F = 46.580, p = 0.000]. The expression of LepRb protein was reduced in the CUMS, Ob and Co groups compared with the Ctr group in the hippocampus (p = 0.000, respectively). The Co group had lower expression of LepRb protein in the hippocampus than the CUMS and Ob groups (p = 0.000, p = 0.026), indicating that stress reduced the expression of LepRb protein in the hippocampus. Figure 5B shows the expression of LepRb protein in the hypothalamus by the Western blot [F (3, 20) = 72.733, p = 0.000]. Main effects of the HFD and CUMS on the expression of LepRb protein in the hypothalamus were significant [F = 130.578, p = 0.000; F = 64.987, p = 0.000], with significant interaction between the HFD and CUMS was found [F = 22.636, p = 0.000]. The expression of LepRb protein was reduced in the CUMS, Ob and Co groups compared with Ctr group (p = 0.030, p = 0.000, p = 0.000). The Co group had lower expression of LepRb protein than the CUMS and Ob groups (p = 0.000, respectively).

### The Effects of HFD and CUMS on the mRNA expression of LepRb

**Figure 6:**
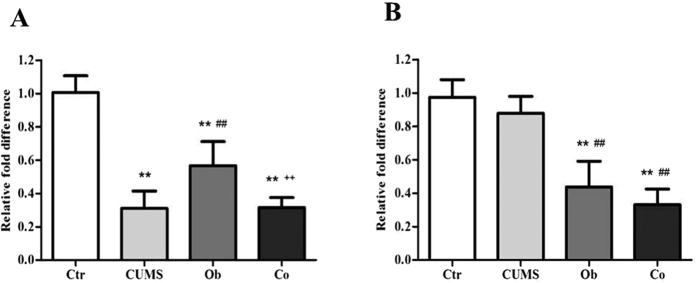
The levels of mRNA expression of LepRb in the hippocampus (**A**) and hypothalamus (**B**) (n = 6 per group). All data are expressed as means ± SD; **P < 0.01 vs. Ctr group; ^##^*P* < 0.01 vs. CUMS group; ^++^*P* < 0.01 vs. Ob group.

Figure 6A shows the mRNA expression of LepRb in the hippocampus [F (3, 20) = 56.612, p = 0.000]. Main effects of the HFD and CUMS on mRNA expression of the LepRb in the hippocampus were significant [F = 24.900, p = 0.000; F = 118.720, p = 0.000], and the interaction between the HFD and CUMS was also significant [F = 26.217, p = 0.000]. The mRNA expression of LepRb in the hippocampus was reduced in the CUMS, Ob and Co groups compared with the Ctr group (p = 0.000, respectively). The CUMS and Co groups had lower expression of LepRb than the Ob group (p = 0.000, p = 0.001). Figure 6B shows the mRNA expression of LepRb in the hypothalamus [F (3, 20) = 45.305, p = 0.000]. Main effects of the HFD and CUMS on mRNA expression of LepRb in the hypothalamus was significant [F = 131.408, p = 0.000; F = 4.490, p = 0.047], but there was no significant interactive effect between the HFD and CUMS [F = 0.015, p = 0.904]. The Ob and Co groups had lower mRNA expression of LepRb in the hypothalamus than the Ctr and CUMS groups (p = 0.000, respectively).

### The correlations between behavioral variables and the levels of LepRb protein and mRNA in the hippocampus and hypothalamus

**Figure 7:**
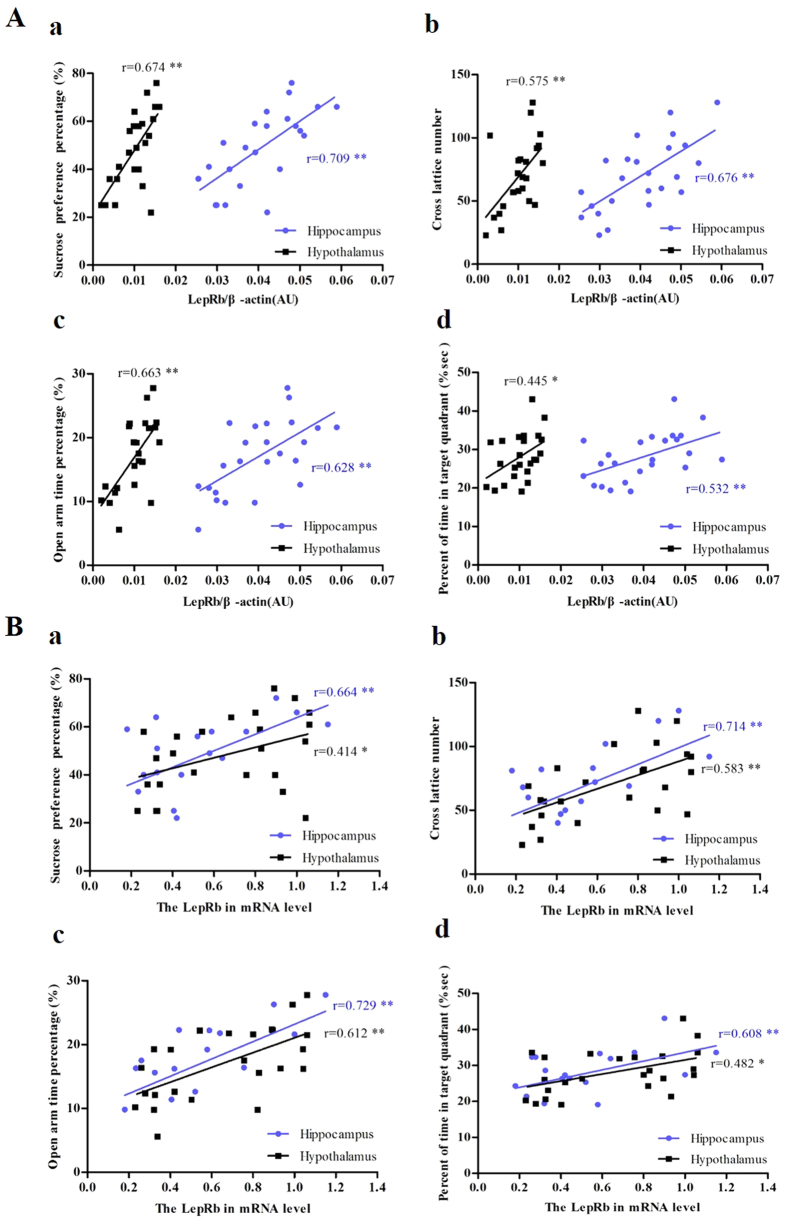
The correlation between behavioral variables and the levels of LepRb protein (**A**) and mRNA (**B**) in the hippocampus (n = 24, 6 per group) and hypothalamus (n = 24, 6 per group). The correlations between sucrose preference percentage (**a)**, cross lattice number (**b**), percentage of time spent in the open arms (**c**), percent of time in target quadrant (**d**) and the levels of LepRb protein and mRNA sepeartly in the hippocampus and hypothalamus were performed by the Pearson’s correlation test. *p < 0.05; **p < 0.01.

Figure 7 shows the correlation analysis between behavioral variables (sucrose preference percentage (a), cross lattice number (b) and percentage of time spent in the open arms (c), as well as spatial learning and memory (percent of time in target quadrant (d)) and levels of LepRb protein (A) and mRNA (B) in the hippocampus and hypothalamus. The results demonstrate significant correlation between behavioral variables and the levels of LepRb protein (sucrose preference: r = 0.706, p = 0.000 in the hippocampus; r = 0.674, p = 0.000 in the hypothalamus; cross lattice numbers: r = 0.676, p = 0.000 in the hippocampus; r = 0.575, p = 0.003 in the hypothalamus; percentage of time spent in the open arms: r = 0.628, p = 0.001 in the hippocampus; r = 0.663, p = 0.000 in the hypothalamus and percent of time in target quadrant r = 0.045, p = 0.007 in the hippocampus; r = 0.532, p = 0.026 in the hypothalamus) and mRNA (sucrose preference: r = 0.664, p = 0.000 in hippocampus; r = 0.414, p = 0.045 in hypothalamus; cross lattice numbers: r = 0.714, p = 0.000 in hippocampus; r = 0.583, p = 0.003 in hypothalamus; open arm time percentage: r = 0.729, p = 0.000 in hippocampus; r = 0.612, p = 0.002 in hypothalamus and target quadrant time percent r = 0.608, p = 0.002 in hippocampus; r = 0.482, p = 0.017 in hypothalamus) in the hippocampus and hypothalamus.

## Discussion

This study described for the first time a model of comorbid obesity and depression in male rats. This model shares common features with humans. The effects of HFD and CUMS on depression-like and anxiety-like behavior, leptin/LepRb signaling of the rats and their interaction impact were investigated. The results of this study were summarized and found in Supplementary Table S1. We confirmed that the HFD could induce obesity and CUMS could cause depression-like and anxiety-like behavior as well as cognitive impairment in rats. This is the first study that reported the interaction effects between adipose/body weight, levels of plasma leptin and LepRb protein in the hippocampus and hypothalamus when the animals were exposed to combined the HFD and CUMS. The correlation analysis showed a significant and positive correlation between depression/anxiety-like behavior, cognitive impairment and the levels of LepRb protein and mRNA in the hippocampus and hypothalamus. Our results were in agreement with the conclusion that leptin and LepRb play an important role in the pathophysiology of obesity and depression^23^,^25^,^31^.

Epidemiological studies have shown that consumption of high energy diet is an important etiology for obesity. This observation was found in animal studies with chronic intake of HFD^32^,^33^,^34^. The present study confirmed that 11-week consumption of HFD increased the body weight and adipose/weight ratio significantly. During the 3-week of CUMS, there was reduction in the body weight of the CUMS group and this finding is congruent with previous studies^35^,^36^,^37^. Nevertheless, body weight and adipose level of the Co group were slightly increased during the CUMS procedure and lower than the Ob group but higher than the CUMS group at the 11^th^ week. These results were consistent with the finding that animals with HFD-induced obesity demonstrated a sudden reduction of food consumption in the first 3 weeks of the CUMS procedure^38^. Interestingly, the CUMS group exhibited a decline in body weight in the first week of CUMS while the rats in Co group demonstrated a small increase in body weight. Therefore, we speculate that the CUMS has mitigating effect on weight gain and increase in adipose tissues. Furthermore, statistical analysis results confirmed the significant interactive effect between CUMS and HFD.

Anhedonia and reduction in explorative interest are considered as important symptoms of depressive disorder which are also found in patients suffering from anxiety disorder^39^. The present study found that the CUMS had reduced sucrose preference during the SPT (i.e. more anhedonia), lower cross lattice number (i.e. less explorative interest) during the OFT and lower percent of time spent in the open arms (i.e. more anxiety-like behavior) during the EMT, which are consistent with former studies^40^. Recent studies reported chronic HFD intake led to depressive- and anxious-like behaviors^38^,^41^, but emotional disturbances were not further aggravated when the HFD animals were exposed to the CUMS^38^. Del Rio *et al*. further reported that HFD could induce antidepressant-like effect but had no effect on anxiety^42^. Our study showed different results because the Ob group exhibited depressive-like and anxiety-like behavior without any antidepressant-like effect. In our study, the Co group did not exhibit higher levels of anhedonia as compared with the CUMS group. Nevertheless, the Co group demonstrated the lowest levels of explorative interest, highest level of anxiety-like behavior and lowest level of spatial learning and memory among the 4 groups. Hence, our results suggest that the combination of obesity and chronic stress will significantly worsen explorative interest, anxiety-like behavior and impairments in spatial learning and memory but not anhedonia as compared to the effect of obesity or chronic stress alone.

Previous studies have reported cognitive impairment and memory deficits induced by CUMS^43^,^44^. In this study, the CUMS group also exhibited significant impairment in spatial learning and memory during the MWM test. Previous research have reported that chronic HFD intake (e.g. for 4 months) had a deleterious effect on the hippocampal-dependent plasticity and memory, particularly in juvenile mice^45^,^46^,^47^. On the other hand, short term exposure to HFD did not exert any effect on the hippocampal-dependent memory when administered to adult mice. In our study, Ob rats didn’t show significantly memory impairment in MWM which may be related with the deficiency of HFD consumption period. The Co group showed the lowest percent of time spent in target quadrant compared with the other groups. This indicated that significant impairments in spatial learning and memory were caused by combined exposure to CUMS and HFD.

Leptin is an anti-obesity hormone which plays a role in satiety regulation. The elevated leptin levels at discharge from the hospital were found to be associated with the risk of developing depression after stroke^48^. The elevated leptin level could be a response to obesity which is a risk factor for stroke. Ge *et al*. reported that the serum levels of leptin and mRNA expression of LepRb were both decreased in depressed rats^49^. In contrast, Morris *et al*. reported that depressed patients had higher levels of serum leptin compared with healthy people. Patients suffering from moderate to severe depression had higher levels of leptin than those suffering from mild depression. Our study found that levels of leptin of the CUMS, Ob and Co groups were significantly higher than the Ctr group. Our results suggest either HFD or CUMS could induce higher levels of plasma leptin. This study further found that there was interaction effects between the HFD and CUMS on plasma level of leptin, which indicated that chronic stress could reverse increased leptin level in rats exposed to HFD. As a result, the Co group did not demonstrate highest levels of leptin among the 4 groups.

Previous studies highlighted that abnormality in the hippocampus in the pathophysiology of emotional disorders and cognitive impairment, and deletion of LepRb in the hippocampus of adult mice could induce depression-like behaviors^50^. This present study further investigated the central LepRb expression in the hippocampus of rats. The expression of LepRb protein and mRNA in the hippocampus of the CUMS, Ob and Co groups was significantly lower than the Ctr group. Moreover, the Co group exhibited lower expression of LepRb protein than CUMS group and Ob group in the hippocampus and hypothalamus. One possible explanation is that chronic stress induced apoptosis and necrosis in hippocampal neurons could lead to mood dysregulation and cognitive impairment^51^,^52^,^53^. Our study also observed the neuronal necrosis in CA1 and CA3 regions of the hippocampus, which support previous findings that chronic unexpected mild stress and chronic social defeat stress could reduce proliferation of neural progenitor cells in the hippocampus (see Supplementary Fig. S1)^54^. Furthermore, there were interactive effects between the HFD and CUMS on the expression of LepRb protein and mRNA in the hippocampus. Depressive and anxiety-like behaviors and memory impairment significantly correlated with the levels of LepRb protein and mRNA in the hippocampus. This implied that the down-regulation of LepRb signaling in the hippocampus might be associated with depressive and anxiety-like behaviors and cognitive impairment in rats.

The hypothalamus is involved in regulation of food intake, body temperature and emotion. Dysfunction of the hypothalamus was found to be closely related to the pathophysiology of depressive disorder^30^. The levels of expression of LepRb protein and mRNA in the Ob group and Co group were significantly lower than the Ctr group in the hypothalamus. More importantly, this study was the first to report that there was interactive effect between the HFD and CUMS on the expression of LepRb protein in the hypothalamus. The depressive-like behaviors and memory impairment were associated with the lower levels of LepRb protein and mRNA in the hypothalamus. These results suggest that lower levels of LepRb expression in the hippocampus and hypothalamus caused by CUMS and obesity alone or in combination could induce depressive and anxiety-like behaviors and cognitive impairment. Our finding indicate that lower levels of LepRb in the hippocampus and hypothalamus may play a critical role in both obesity and comorbid depression and the specific mechanisms causing these phenomena require further study.

## Methods

### Animals

Sixty-eight male Wistar rats purchased from the Experimental Animal Center of Shandong University, which were 9 weeks old and weighing 200–230 grams, were housed in groups of 4–6 and maintained under 12 hrs light-dark cycle, at 20–24 °C with free access to food and water. All procedures used in this study were reviewed and approved by the Ethics Committee of the Medical school of Shandong University, which complied with the National Institute of Health Guide for the Care and Use of Laboratory Animals (NIH publication No. 85-23, revised 1985).

### Experimental procedure

After 7 days of adaptation, rats were randomly divided into 2 groups according to body weight. 24 rats fed with regular diet were in the regular diet group (RD, 8% fat, Animal Center of Shandong University) while 44 rats fed with high-fat diet were in high-fat diet group (HFD, 60% of calories derived from fat, 20% of calories derived from protein, 20% of calories derived from carbohydrate; Research Diets of New Brunswick D12492, NJ.). Food intake of rats was recorded every day by weighing uneaten food each morning and body weight of all rats was recorded per week. At the end of the 8th week, 26 rats whose body weights were over the average weight of +1.96 times the standard deviation of the regular diet group were assigned into the diet-induced obesity (DIO) group. Subsequently, rats in RD group and DIO group were randomly divided into unstressed groups and stressed groups. Therefore, there were four groups in the study: control group (Ctr, n = 12), CUMS group (CUMS, n = 12), obesity group (Ob, n = 13), combined obesity and CUMS group (Co, n = 13).

### Chronic unpredictable mild stress

Rats in the CUMS group and Co group were repeatedly exposed to a series of chronic unpredictable mild stressors that included the following: 8 hours of food deprivation, 8 hours of water deprivation, 45° leaning of the rat’s cage, group feeding (Two cages were combined), confusing day and night, soaking the cage with water, and horizontal oscillation for 20 minutes. Each stressor was applied every day, and the entire stress procedure lasted for 3 weeks, with stressors applied in a completely random order inducing a depressive state^55^.

### Behavior tests

#### Sucrose preference test (SPT)

A decreased preference for sucrose has been considered to be homologous to anhedonia, one of the defining symptoms of major depression^55^,^56^. Prior to the test, there was a 4-days adaptation period for rats to habituate to the 1% (W/V) sucrose solution or water. After that, a 23-hour period of water and food deprivation was carried out. Then each rat was put into one cage separately and was given free access to two bottles for 1 hour, one with 200 ml sucrose solution and the other with 200 ml water. The sucrose preference percentage was evaluated by the amount of sucrose solution consumed among all fluids consumption.

#### Open-field test (OFT)

Open field test was used to test the exploration motivation of rats^57^. Rats were placed individually in the center of the apparatus and left freely to explore the arena for 5 minutes. Total cross lattice number and total cross distance were recorded by a camera that was linked to a computer that was fitted with SMART video tracking system.

#### Elevated plus maze test (EMT)

The elevated plus maze consisted of 4 arms designed in a cross-shape, of which 2 were open and 2 were close. The 4 arms meet in a 5 cm × 5 cm central square open area which enabled the rats to freely enter each arm of the maze. Rats were placed in the central square area of the maze facing an open arm and allowed to freely explore the maze for 5 minutes. Movement of the rats in the maze was recorded by a camera linked to a computer. Time spent in the open arms, number of times of entering the open arms and distance traveled in each arm by the rats were analyzed^58^. The lower percentage of time spent in the open arms indicates more anxiety-like behavior in rats.

### Morris Water Maze

Spatial learning and memory of the rats were assessed by the MWM^59^. Rats were allowed to swim freely for 60 s every 24 hours before the formal training. During the following 4 consecutive days, the rats were trained to find a platform (15 cm diameter) hidden under the water surface in the quadrant IV 4 times per day. In each training, the rats were put in the water facing the pool wall from one of the four points spaced 90° around the border of pool and given a 120 second swimming time limit. If the rats could find the platform, they were required to remain on the platform for 15 seconds. Otherwise, they were guided to the platform and had to remain on it for 15 seconds. On the 5^th^ day, rats were released into the maze without the hidden platform from an identical point of quadrant I and allowed to swim freely for 60 seconds. The percentage of time rats spent in quadrant IV on the 5^th^ day was analyzed.

### Tissue preparation

24 hours after MWM, all rats were weighted. Then, 8 rats of each group were anesthetized with pentobarbital and euthanized by decapitated. The brains were removed rapidly and dissected on ice. The hippocampus and the hypothalamus were quickly isolated and dipped into liquid nitrogen and stored at −80 °C for RNA and protein extraction. Blood was gathered into anticoagulation tubes and 3000 r/min low temperature centrifugation to collect plasma. Fat of Retroperitone, omenta and epididymis were measured. Other rats were anesthetized with pentobarbital and perfused with 50 ml of 0.1 m PBS, followed by 200 ml ice-cold 4% paraformaldehyde (PFA). The brains were removed and immersed in the PFA for 24 hours. The fixed brain was dehydrated and embedded in paraffin.

### Measurement of adipose/weight

Adipose/weight percent (%) was calculated as ([retroperitoneal fat (g) + omental fat (g) + epididymal fat (g)]/[body weight (g) at the end of experimental procedure]) × 100^60^.

### Plasma leptin levels

Plasma concentrations of leptin were determined by using rat leptin ELISA kits (Huijia Biotechnology Company, Xiamen, China).

### Immunohistochemistry

Paraffin-embedded brain tissues were cut to a thickness of 5 μm. Sections were deparaffinized with xylene and rehydrated with a graded alcohol series, followed by 9 minutes of heat-induced antigen retrieval in a microwave in a sodium citrate buffer (pH 6.0) and allowed to cool for 45 minutes at room temperature. Endogeous peroxidase activity was blocked with 3% hydrogen peroxide for 10 min. After washing with phosphate-buffered saline (PBS) for 3 times, slides were incubated with 10% bovine serum albumin for 15 minutes. Thereafter, the slides were incubated with the primary polyclonal antibody for LepRb (sc-8325, Santa Cruz Biotechnology, Inc., dilution 1:250) overnight at 4  °C. After rinsing in PBS for 3 times, the slides were incubated with the secondary antibody and the streptavidin/HRP complex for 30 minutes at 37 °C respectively. Diaminobenzidine/H_2_O_2_ was used as the chromogen for 4 minutes and hematoxylin as the counterstain for 10 minutes. Five high-power fields were randomly selected subfields of hippocampus and hypothalamus at 400× magnification. LepRb expressive cell counts were performed.

### Protein extraction and Western Blotting

Hippocampus and hypothalamus (30 mg, separately) were homogenized on ice with 2 mM phenylmethanesulfonyl fluoride in 1 ml ice-cold RIPA buffer added protease inhibitor. The protein concentration in the supernatant was harvested and determined by the BCA analysis. Equal amounts of protein (40 ug) from each sample were separated by 12% SDS-PAGE and transferred to PVDF membranes (Millipore, Boston, MA). The membranes were blocked by non-fat dry milk buffer for 1 hour and then immunoblotted overnight at 4 °C with primary antibody against LepRb (sc-8325, Santa Cruz Biotechnology, dilution 1:250) and β-actin (Proteintech, USA; dilution 1:1000). After being washed for three times, the membranes were incubated with HRP-conjugated secondary antibody (Proteintech, USA; dilution 1:2000) for 1 hour at room temperature, washed and visualized by employing enhanced chemiluminescence. The optical densities of bands were recorded and analyzed by using C-Digit (LI-COR, USA). Relative expression was normalized to β-actin.

### RNA extraction, reverse transcription cDNA synthesis and quantitative PCR

Total RNA was extracted from homogenized tissues using Trizol (Invitrogen, Carlsbad, CA, USA). Reverse transcription and real-time quantitative PCR of total cDNA were performed using standard reagents and protocols (CWBIO, Beijing, China). The real-time quantitative PCR was used in 96-well format with a 20 μl reaction volume per well, which was performed on an ABI Prism 7500 sequence detection system. In general, PCR was performed at 95 °C/5 minutes, forty cycles were run with the following conditions: 95 °C/30 seconds, 60 °C/30 seconds, 72 °C/30 seconds and terminated at 72 °C/5 minutes. The amount of mRNA was normalized to the expression of β-actin. All PCR data analysis used the 2^−ΔΔ^CT method.

### Statistics Analysis

Results were presented as mean ± SD and were analyzed by SPSS16.0. The data were tested by two-way ANOVA of 2 × 2 factorial design followed by LSD post hoc test for grouped comparison. The Pearson’s correlation test was used to analyze the correlation between the behavioral variables and LepRb Level. Differences were considered significant when a minimum value of P less than 0.05.

## Additional Information

**How to cite this article**: Yang, J. L. *et al*. The Effects of High-fat-diet Combined with Chronic Unpredictable Mild Stress on Depression-like Behavior and Leptin/LepRb in Male Rats. *Sci. Rep.* **6**, 35239; doi: 10.1038/srep35239 (2016).

## Supplementary Material

Supplementary Information
