# Supplementary material for: The Effects of High-fat-diet Combined with Chronic Unpredictable Mild Stress on Depression-like Behavior and Leptin/LepRb in Male Rats
Source: Sci Rep. 2016 Oct 14;6:35239. doi: 10.1038/srep35239 (PMC5064321; doi:10.1038/srep35239)
Supplement: Supplementary Information [file srep35239-s1.doc]

**The Effects of High-fat-diet Combined with Chronic Unpredictable Mild Stress on Depression-like Behavior and Leptin/LepRb in Rats**

**Authors:** Jin Ling Yang MD^1^, De Xiang Liu PHD ^1^, Hong Jiang BD^1^, Fang Pan PHD ^1*^, Su Hui Ho MBBS, MRCPsych^2^, Roger CM Ho MBBS, FRCPC ^2^

1 Department of Medical Psychology, Shandong University School of Medicine, Jinan, Shandong, China, 250012

2 Department of Psychological Medicine, National University of Singapore, Singapore, 119228

***Correspondence:** Professor Fang Pan, Department of Medical Psychology, Shandong University School of Medicine, 44#, Wenhua Xi Road, Jinan, Shandong, 250012, China. Email: panfang@sdu.edu.cn*.*

***Supplementary*** ***Table s1***

|  | Group comparison | The highest and lowest |
| --- | --- | --- |
| Adipose / Weight % at the 11^th^ week | Ob > Co > Ctr > CUMS | Ob -The highest adipose / weight %  CUMS -The lowest adipose / weight % |
| Depression-like behavior | Co > CUMS > Ob > Ctr | Co -The most severe depression-like behavior  Ctr -The least severe depression-like behavior |
| Leptin | Ob > CUMS > Co > Ctr | Ob - The highest leptin level  Ctr - The lowest leptin level |
| LepRb expression in the hippocampus | Ctr > Ob > CUMS > Co | Ctr - The highest LEPR expression  Co - The lowest LEPR expression |
| LepRb expression in the hypothalamus | Ctr > CUMS > Ob > Co | Ctr- The highest LEPR expression  Co - The lowest LEPR expression |
| mRNA expression of the LepRb in the hippocampus | Ctr > Ob > CUMS > Co | Ctr- The highest mRNA LEPR expression  Co - The lowest mRNA LEPR expression |
| mRNA expression of the LepRb in the hypothalamus | Ctr > CUMS > Ob > Co | Ctr - The highest mRNA LEPR expression  Co - The lowest mRNA LEPR expression |

**Supplementary Figure 1**


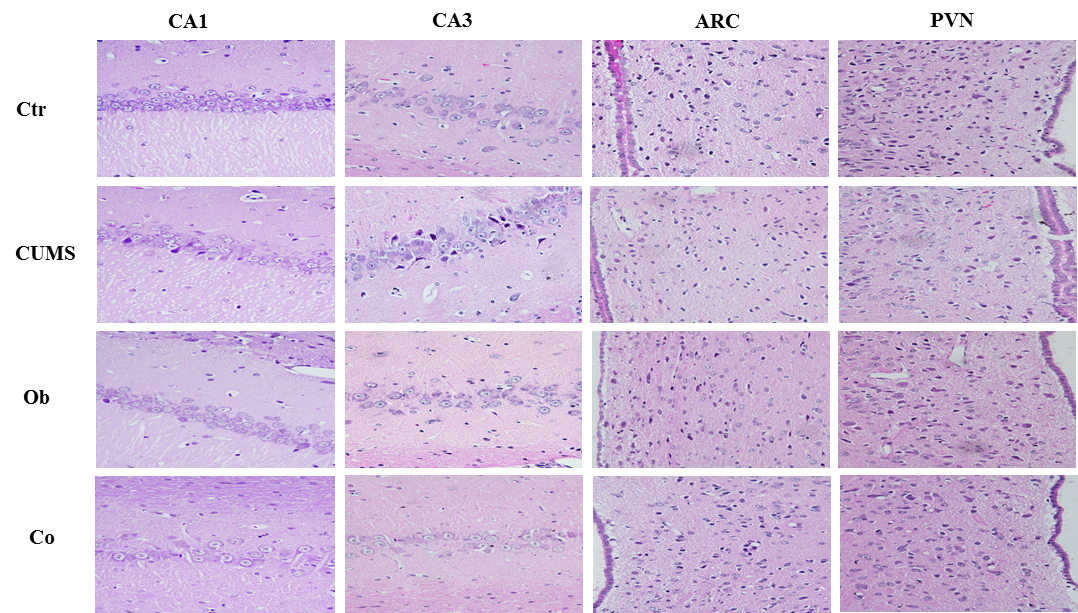


Morphology of the hippocampus cells (CA1 and CA3) and hypothalamus cells (ARC and PVN) (n=4～5/group, hematoxylin-eosin staining, × 40)
